# Decentralized Trusted Data Sharing Management on Internet of Vehicle Edge Computing (IoVEC) Networks Using Consortium Blockchain

**DOI:** 10.3390/s21072410

**Published:** 2021-03-31

**Authors:** Muhammad Firdaus, Sandi Rahmadika, Kyung-Hyune Rhee

**Affiliations:** 1 Department of Artificial Intelligence Convergence, Pukyong National University, Busan 48513, Korea; mfirdaus@pukyong.ac.kr (M.F.); sandika@pukyong.ac.kr (S.R.); 2 Department of IT Convergence and Application Engineering, Pukyong National University, Busan 48513, Korea

**Keywords:** IoV, blockchain, trust management, smart contracts, PBFT, incentive mechanism

## Abstract

The emergence of the Internet of Vehicles (IoV) aims to facilitate the next generation of intelligent transportation system (ITS) applications by combining smart vehicles and the internet to improve traffic safety and efficiency. On the other hand, mobile edge computing (MEC) technology provides enormous storage resources with powerful computing on the edge networks. Hence, the idea of IoV edge computing (IoVEC) networks has grown to be an assuring paradigm with various opportunities to advance massive data storage, data sharing, and computing processing close to vehicles. However, the participant’s vehicle may be unwilling to share their data since the data-sharing system still relies on a centralized server approach with the potential risk of data leakage and privacy security. In addition, vehicles have difficulty evaluating the credibility of the messages they received because of untrusted environments. To address these challenges, we propose consortium blockchain and smart contracts to accomplish a decentralized trusted data sharing management system in IoVEC. This system allows vehicles to validate the credibility of messages from their neighboring by generating a reputation rating. Moreover, the incentive mechanism is utilized to trigger the vehicles to store and share their data honestly; thus, they will obtain certain rewards from the system. Simulation results substantially display an efficient network performance along with forming an appropriate incentive model to reach a decentralized trusted data sharing management of IoVEC networks.

## 1. Introduction

With the rapid movement of urbanization and industrialization, the number of registered vehicles worldwide is estimated to reach two billion within the next 10–20 years [1]. It will bring multiple challenges for the future transportation system. The intelligent transportation system (ITS) framework has gained expanding enthusiasm from academia and industry as the solution to address these challenges. ITS is expected to compose an indispensable part of developing smart cities in the vehicular network context by leveraging the internet of vehicles (IoV) concept. Hence, IoV that combines smart vehicles and the internet is a key enabler technology that facilitates the next generation of ITS. The IoV allows vehicles to share road-related information messages with their neighbors, e.g., road conditions, traffic congestions, accident information, and safety warnings. Consequently, vehicles can be more aware of traffic situations, as well as contribute to improving the system transportation safety and efficiency [2].

However, the conventional IoV system has difficulty overcoming the increasing complexity of ITS applications that exponentially led to the demand for the enormous data storage volumes with high computation and communication processing requirements. Hence, the notion of IoV edge computing (IoVEC) is introduced to enhance user experience with low latency, high bandwidth, and real-time communications with the help of mobile edge computing (MEC) technology [3]. IoVEC is defined as integrating vehicular networks and MEC that offers various opportunities to support massive data storage and provide computing process close to vehicles as a data provider. Moreover, IoVEC aims to reduce the communication overhead and support the delay-sensitive application by placing its computing resource in the edge network near the vehicular network user. Therefore, IoVEC allows the system to minimize service delay, achieve low transmission latency, alleviates network congestion, and enhances the quality of service (QoS) [4].

Despite the potentials mentioned above, the conventional architecture of IoVEC with a centralized approach has crucial challenges related to user’s data security and privacy. In this sense, the potential exposure of user information with single point of failure (SPoF) challenges still will reasonably occur since the IoVEC framework’s data is centralized on a central server. Hence, the IoV network participants might be hesitant in the data sharing process that contains private information, such as customer identities, vehicle numbers, and driving preference. Moreover, the risk of selfish behaviors might diminish participants’ enthusiasm to cooperate with each other in the system. This problem becomes more serious when there exists a malicious vehicle in the network. The various adversarial actions may threaten the privacy security to gather the user’s private information for personal benefit, as well as endanger traffic safety and efficiency, by giving the incorrect report to the system. Consequently, IoVEC needs to construct a reliable and trusted data sharing management model by removing a centralized intermediary scheme.

Previous work considers that vehicle may share their data voluntarily [5]. Unfortunately, it might cause the low participation of vehicles in the data sharing process and then affects the system’s reliability in the future. Moreover, due to self-interest characteristics, vehicles may also be unenthusiastic to share the data because they do not obtain particular benefits or compensation from the system. Therefore, some forms of incentive mechanism should be provided to motivate honest vehicle (as data owner) to give a relevant contribution with long-term participation for system reliability and sustainability. Incentive mechanism provides rewards to the vehicles that contribute to the data sharing process to form the trusted data management system in IoVEC. However, existing incentive mechanisms generally use a central trusted authority to orchestrate the system, such as monetary-based incentive [6] and reputation-based incentive [7]. In this sense, a central trusted authority that holds the user data poses a potential risk where a single mistake might affect the entire system’s orchestration. Therefore, the need to form fair incentives without involving the central trusted authority is vital for a secure and trusted data management system in IoVEC networks.

In recent years, since Nakamoto introduced Bitcoin [8] in 2008, blockchain as an emerging technology has gained much attention to cope with security and privacy issues because of its anonymity, decentralization, and trust characteristics. Basically, blockchain combines the advantages of peer-to-peer (P2P) networks and cryptography algorithms to ensure the transaction agreements’ validity among involved participants without intermediary authority. These recorded transactions are secure and cannot be altered maliciously after being added into an immutable distributed database. Hence, currently, blockchain with its various advantages has been applied in various fields [9], such as financial industry [10], healthcare industry [11], smart energy system [12], and industrial Internet of Things [13]. In the vehicular network context, blockchain is widely studied to form a secure, trusted, and decentralized ITS [14]. Several works proposed blockchain-based IoV to protect sharing road-related information among vehicles to improve traffic safety and efficiency [2,15,16]. Motivated by these developments, we utilize a consortium blockchain and leverage the smart contracts to develop a decentralized trusted data sharing management system in IoV. First, we design an information credibility assessment scenario to minimize irrelevant information data. The system allows the vehicle to assess the received information’s credibility from its neighboring vehicles in the same region by generating the specific rating. Second, we use the practical byzantine fault tolerance (PBFT) for consensus mechanism since it is suitable for consortium blockchain [17] as our proposed model framework. In the consortium blockchain, multiple preselected nodes (e.g., selected by the consortium members under the Department of Transportation supervision) are authorized to perform the consensus process to validate all transactions of the shared data before appending them into a distributed ledger database [18]. The roadside units (RSUs) are then defined as the preselected edge nodes and placed along the road, which plays a crucial role in providing a trusted data sharing management system in IoVEC. Third, we also present an appropriate incentive mechanism based on blockchain to trigger vehicles to participate positively to improve and maintain the system’s reliability and sustainability. Concisely, the contributions of this paper are summarized as follows.

1. We design a decentralized trusted data sharing management framework for IoVEC networks by utilizing a consortium blockchain and smart contracts. This framework proposes a secure data sharing scenario among vehicles without relies on trusted intermediaries in distributed, verifiable, and immutable ledgers.
2. We present an information credibility assessment scenario to minimize irrelevant information data and against malicious behaviors of the vehicle on the data sharing process.
3. We design an appropriate incentive mechanism based on the vehicle’s contribution by leveraging smart contracts’ self-execution nature. This scheme aims to motivate vehicles to participate positively in maintaining trusted data sharing activities and ensuring the system’s security and sustainability.
4. We formulate a decentralized data sharing of IoV networks prototype and evaluate its performance based on simulation results.

The remainder of this paper is organized as follows. Section 2 describes the problem definition based on traditional data sharing management on the conventional vehicular network with a centralized incentive mechanism. Related work is presented in Section 3. Then, we explain the design architecture of the IoV-blockchain, including its detailed procedures in Section 4. We demonstrate the proposed design by analyzing its performance in Section 5. Finally, Section 6 concludes the paper.

## 2. Related Work

In this section, we review relevant literature on conventional data sharing management on vehicular network and centralized incentive mechanism to position the existing approach in relation to our research.

### 2.1. Internet of Vehicle Edge Computing (IoVEC) Networks

Mobile edge computing (MEC) technology is introduced in 2014 by the European Telecommunications Standards Institute (ETSI), aiming to heighten user experiences with low latency, high bandwidth, and real-time communications [3]. MEC leverages the local server infrastructure to reduce response and delay time during the transaction process by placing its server in the edge network to be closer to the user. Encouraged by the advantages of MEC, many works have been dedicated to combining vehicular network (VN) into MEC, thereby establishing vehicular edge computing (VEC) networks, where an extra edge infrastructure is the main distinction that differentiates between conventional VN and VEC. In Reference [19], the authors formulated an offloading problem with joint load balancing in VEC to enhance system utility and network effectiveness. Moreover, they also proposed a low-complexity algorithm to optimize the decision-making on computation resource and offloading ratio selection. The authors in Reference [20] offered a novel mechanism, namely mixed-integer nonlinear programming (MINP) formulation, to minimize resource sharing expense while enhancing the quality of service (QoS) indicators. In Reference [21], the authors concentrated on providing an efficient distributed reputation management for VEC networks. The internet of vehicles edge computing (IoVEC) networks can be considered the extension of VEC in the IoV environment, which works in a similar fashion to the traditional VEC networks.

### 2.2. Conventional Data Sharing Management

In the vehicular network environment, the primary entities in the data sharing process are vehicles and roadside units (RSUs), which form two types of communication, namely vehicle-to-vehicle (V2V) and vehicle-to-infrastructure (V2I). Vehicles interact with other neighboring vehicles by using onboard units (OBUs) equipped with several sensing devices with simple computation and communication capabilities. OBUs are also used to automatically recognize traffic-related information and facilitate the vehicles to send notification messages to others using V2V communication standards to improve traffic safety and efficiency. On the other hand, V2I provides a single or multi-hop communication between vehicle and RSU, supported by dedicated short-range communication (DSRC) standards [22]. RSUs, as the roadside infrastructure, provide wireless communications along the road to vehicles. Thus, RSUs are prepared to aggregate the traffic data in a particular coverage area in the VN system.

Moreover, conventional VN data management relies on a cloud service platform with a centralized database approach. In this sense, the centralized server is employed to collect and store all vehicles’ shared data centrally. However, the centralized approach is still facing the threat of security and privacy risk, where the attackers can easily forge or tamper with the data sent by OBUs in an open wireless communication environment [23]. Further, a centralized server might also reveal and trade the user’s private data to personally obtain a particular profit by neglecting user privacy consideration. Therefore, we introduce the decentralized system to cope with the centralized approach problem to facilitate the trusted management system. In this regard, we propose blockchain as the distributed database system which facilitates a transparent data sharing transaction by involving all participants to approve each transaction using a particular consensus mechanism to validate data recorded with time-stamp before stored into an immutable database without the help of a central trusted intermediary.

### 2.3. Centralized Incentive Mechanism

Since the VN system relies on a centralized approach, vehicles might be reluctant to share their data due to data security and privacy protection issues. Moreover, the SPoF is likewise a significant problem for centralized networks. Another challenge is that vehicle participation in the data sharing process remains low due to self-interest characteristics, and the vehicles do not obtain the compensation or benefit from the system. Hence, the incentive scheme is used to boost vehicle participation with positive contribution and maintain the system’s reliability and sustainability. The existing incentive mechanism with a centralized approach allows simple transactions between vehicles and a trusted third party. In this case, a trusted third party holds the entire data transaction and provides transaction incentives among participants involved. In short, as a data provider, the trusted third party controls the whole system orchestration (including incentive scheme).

Current works that represent centralized incentive approach are monetary-based incentive [6] and reputation-based incentive [7]. The monetary-based incentive uses the payment strategy to avoid inefficient contributions and unnecessary rewards while manages payments and charges from a game-theoretic perspective. This scheme motivates participants to report their behavior honestly and receive electronic money as rewards. On the other hand, the reputation-based incentive evaluates the participants’ trustworthiness in certain actions according to their prior experiences using a game-theoretic model based on repeated games. This scheme is designed to provide an appropriate incentive according to participants’ contribution along with identifying uncooperative participants. However, both schemes are still insufficient to be implemented in a trusted data management system due to the centralized approach risks and challenges. It worth noting that in terms of SPoF, a single mistake might alter the entire system’s orchestration. Hence, malicious vehicles easily manipulate the system transactions and obtain all vehicles’ data as long as they control the attacked central server. Further, the centralized server may possibly be congested due to a large number of vehicle transactions resulting in reduced system performance [24]. In this case, we suggest leveraging blockchain-based smart contract to form an incentive mechanism in a decentralized approach to address these issues. This approach aims to improve privacy and security protection, encourage vehicles to participate positively in VN data sharing activities, along with improving the system’s performance.

### 2.4. Blockchain-Based Decentralized Data Sharing Management

Blockchain is considered as a solution to improve security and privacy protection since it is suitable to overcome the centralized problem approach. Blockchain is a distributed ledger technology that enables participator entities to accept and share recorded activities with a time-stamp to the network. The particular consensus mechanism validates those activities before it is filed in a changeless database. Generally, blockchain can be utilized to achieve three objectives: managing distributed services relying on smart contracts, operating a distributed ledger, and accomplishing decentralized storage [25]. There exist many efforts that concentrate on implementing blockchain technology into VN. The authors in Reference [26] reviewed the latest researches of the blockchain-based IoV by classifying research barriers and technical problems. They separate blockchain into three layers: perception, networking, and application layer. The perception layer is utilized to address trusted management problems by achieving an accurate data sharing perception. The networking layer aims to protect network security, while the application layer solves accountability and privacy issues.

Moreover, the authors in Reference [2] proposed a blockchain-based decentralized trust management system in VN. They utilize the Bayesian inference model for message credibility assessment to prevent unrelated or false data from a malicious vehicle. The system enables the nearby vehicle to assess the accepted message by generating the message rating to RSUs. On the other hand, RSUs play as miners to create the block by applying joint proof-of-work (PoW) and proof-of-stake (PoS) consensus algorithm. In Reference [27], the authors also exploit a Bayesian network that quickly identifies fake messages by counting the subsequent probability of an event. The event probability is calculated based on several parameters, such as pre-traffic probability, traffic event period, vehicle honesty, and the number of events provided by vehicles. Further, in Reference [28], the authors proposed blockchain to form a trust authentication mechanism in VN. They focus on the secondary authentication model to form a decentralized autonomous VN system that is more reliable and safe from numerous attacks.

Furthermore, several efforts study employing consortium blockchain’s advantages to minimize malicious entities’ existence during the consensus mechanism. The authors of Reference [15] proposed consortium blockchain and smart contract to build a secure P2P data sharing system in VEC. They manage the vehicle’s reputation scheme using a three-weight subjective logic model. A joint PoW and PoS consensus mechanism is utilized to improve system security and efficiency. In Reference [16], the authors presented a data-sharing framework using a consortium blockchain by applying a smart contract through the preselected nodes to maintain data storing and sharing system. The digital signature technique guarantees the data’s integrity and security during the data sharing process. Further, a joint PoW and PBFT consensus is deployed to validate each block transaction before saving toward an immutable blockchain ledger. On the other hand, the authors in Reference [29] proposed a novel privacy-preserving incentive announcement network using blockchain called CreditCoin. CreditCoin motivates users with incentives to share traffic information based on the PBFT consensus mechanism scheme. In Reference [30], the authors proposed an incentive model to trigger the vehicle in accurate and timely data sharing by leveraging Ethereum smart contract. Furthermore, to verify the data sharing transactions and improve system performance, they employ a consensus mechanism based on Proof-of-Authority (PoA).

## 3. The Framework of IoVEC-Blockchain for Trusted Data Management

**Figure 1:**
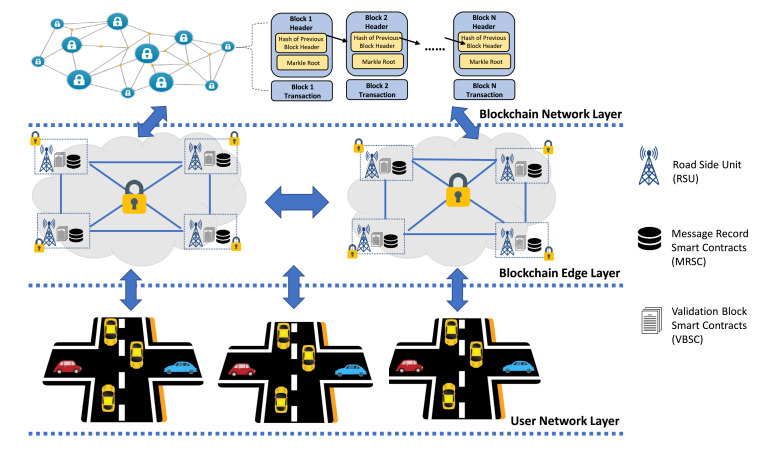
Overview of proposed model.

This section explains our proposed model, blockchain-based secure and trusted data sharing management in IoVEC networks. Inspired by Reference [31], our architecture model relies on consortium blockchain that consists of three layers, as shown in Figure 1. First, the user network layer provides data sharing communication among vehicles as the network user. Second, the blockchain edge layer responsible for validating data sharing transactions. Third, the blockchain network layer provides a decentralized incentive mechanism based on the vehicle’s contribution. Additionally, the proposed model authorizes RSUs as the multiple preselected edge nodes to perform the consensus mechanism. Here, RSUs are distributed along the road to be traffic handlers to control a group of vehicles in a certain radius range. We consider many users (i.e., message provider vehicle and message assessor vehicle) and many validators (i.e., RSUs), both play as the primary entities to form trusted data sharing management systems IoVEC. We use the PBFT consensus mechanism to facilitate strong consistency and a proper consortium blockchain framework. Furthermore, to motivate the vehicle’s contribution, we propose a decent incentive mechanism in a decentralized manner. Table 1 summarizes the notation used to describe our proposed method.The detail system is described as follows.

### 3.1. User Network Layer

User network layer manages vehicle enrollment and authentication, the road-related message broadcasting, and the message credibility assessment process.

#### 3.1.1. Vehicle Initialization and Enrollment

In our scenario, vehicles represent the user network that communicates with other vehicles and RSUs to improve traffic safety and efficiency. Before entering and accessing the network service, all vehicles must be authorized by a trusted party (TP), e.g., the Department of Transportation, to guarantee vehicle identity legitimation by binding their real identities (e.g., vehicle ID or driver’s license). Then, a legitimate vehicle $\left(V_{i}\right)$ that passes the authentication process with real identity creates its public keys $\left(PK_{V_{i}}\right)$, private keys $\left(SK_{V_{i}}\right)$, and certificates $\left(Cert_{V_{i}}\right)$ for securing data sharing transaction. After entering the network, $V_{i}$ downloads the latest data from the nearby edge node’s local data storage (RSU). $V_{i}$ equipped with OBUs and their sensing devices automatically collect road-related messages $M=(M_{1},M_{2},\ldots ,M_{I})$ according to the road occurring events, such as snow reports on the road, weather conditions, traffic jams, safety warnings, and accident information. In IoVEC-blockchain, these messages are encrypted using the elliptic curve digital signature (ECDSA) algorithm as the asymmetric cryptography to ensure communication security and identity anonymity, such as used in Reference [25]. The system enforces the user to use a new address for every new data-sharing transaction. Using this many-addresses scenario, the user’s ownership will be more difficult to trace, thus preserving identity anonymity.

#### 3.1.2. Message Credibility Assessment

In collecting $M_{i}$, $V_{i}$ is helped by OBUs that consists of sensor devices, a memory unit, and a communication module to form simple computation and communication. In the message credibility assessment process, $V_{i}$ plays different roles: as a message provider $\left(V_{P}\right)$ and a message assessor $\left(V_{A}\right)$. With the help of OBUs, $V_{P}$ collects $M_{V_{P}}$ at a specific location *k* and time *t* and encrypts those messages before being broadcasted to the network by utilizing V2V and V2I communication. Nevertheless, $V_{P}$ might behave as a dishonest vehicle and endanger traffic safety and efficiency by giving the incorrect report of $M_{V_{P}}$ to the system. Hence, the system allows the nearby vehicles with the occurred events $M_{V_{P}}$ to be the message assessor $V_{A}$ and evaluate the credibility of $M_{V_{P}}$. $V_{A}$ divides all messages into groups $\left(G_{m}^{1},G_{m}^{2},\ldots G_{m}^{k},\ldots \right)$, where $G_{m}^{k}$ represent the $M_{i}$ in the event location *k*. We consider that the message sent by vehicles near the event location is more trusted compared to the vehicle in a far distance. Therefore, the message credibility is defined based on Equation (1).

(1) $$c_{p}^{k}=\beta +M_{t,P}^{k^{-\gamma .d_{p}^{k}}},$$

where

(2) $$M_{t,P}^{k}=(M_{V_{P}}||timestamp||location).$$

From Equation (1), $c_{p}^{k}$ is the message credibility of $V_{P}$ in group $G_{m}^{k}$, while $d_{p}^{k}$ is the distance among the occurred event in location *k* and the message provider $V_{P}$. There are two predefined parameters: γ represents a criterion that influences the rate of $c_{p}^{k}$ based on $d_{p}^{k}$, while β is the message rating’s lower bound [2]. After calculating $c_{p}^{k}$, $V_{A}$ obtains a credibility set for $M_{V_{P}}$ which then result in $C^{k}=\left(c_{1}^{k},c_{2}^{k},c_{3}^{k},\ldots c_{N}^{k}\right)$. Hence, $V_{A}$ can calculate the aggregated credibility of $M_{V_{P}}$ according to the credibility set $C^{k}$ using Bayesian Inference [32] by the following equation:

(3) $$P\left[M_{V_{P}}|C^{k}\right]=\frac{P\left[M_{V_{P}}\right]\prod_{P=1}^{N}P\left[c_{p}^{k}|M_{V_{P}}\right]}{\sum_{h=1}^{I}\left(P\left[M_{h}\right]\prod_{P=1}^{N}P\left[c_{p}^{k}|M_{h}\right]\right)},$$

where $P[M_{V_{P}}]$ is the prior probability of $M_{V_{P}}$, and $P\left[c_{p}^{k}|M_{V_{P}}\right]=c_{p}^{k}$. We consider $M_{h}$ as the complementary of $M_{V_{P}}$; thus, $P\left[c_{p}^{k}|M_{h}\right]=1-c_{p}^{k}$. Here, the range value of $P\left[c_{p}^{k}|M_{V_{P}}\right]\in \left[0,1\right]$, whereas if $c_{p}^{k}=0$, it represents that $V_{P}$ does not report $M_{V_{P}}$. Then, $V_{A}$ generates the rating based on $P[M_{V_{P}}|C^{k}]$, defined by a certain threshold: a positive rating (+1) for the correct messages, otherwise a negative rating (−1). For example, let the aggregated message credibility yield the value of 0.75. If the threshold is defined as 0.51, the system will regard messages reporting the event as correct and give positive ratings (+1) to the corresponding vehicles. For messages stating otherwise, the reporting vehicles will be given negative ratings (−1). Accordingly, the rating of $M_{V_{P}}$ is uploaded by $V_{A}$ to a nearby edge node with format $M_{V_{A}}$ = $\left(V_{P},V_{A},M_{t,P}^{k},\phi_{P_{A}}\right)$, with $\phi_{P_{A}}$ refers to the rating value, where $\phi_{P_{A}}$∈ [−1,1]. Therefore, the main activities in the user network layer are: $V_{P}$ periodically transmits $M_{V_{P}}$, while $V_{A}$ evaluates the credibility of the $M_{V_{P}}$ by generating and uploading $M_{V_{A}}$ to blockchain edge layer.

### 3.2. Blockchain Edge Layer

Blockchain edge layer plays a critical role in forming a trusted data sharing management system in IoVEC, including message aggregation, consensus mechanism, and block generation.

#### 3.2.1. Message Aggregation and Vehicle’s Reputation

The RSUs are the edge nodes infrastructure and traffic handlers distributed along the road to manage the vehicle network layer’s data sharing process. In our proposed model, we design RSU equipped with two types of smart contracts: message record smart contract (MRSC) and validation block smart contract (VBSC). MRSC collects, records, and aggregates the number of message information (i.e., $M_{V_{P}}$ from $V_{P}$ and $M_{V_{A}}$ from $V_{A}$) from the user network layer in a distributed framework, whereas VBSC stores the resulted data from MRSC into a blockchain network layer. It is worth noting that MRSC records all the participants, i.e., $V_{P}$ and $V_{A}$, which contribute to the data sharing process. Once $V_{A}$ uploads the message ratings $M_{V_{A}}$ into MRSC, the nearby RSU validates the message $M_{t,P}^{k}$ by calculating the aggregation of trust value rating using the majority rule. Here, we assumed that malicious vehicles could not control most of the vehicles in the network. Thus, the trust value rating $\left(\psi_{P}^{t,k}\right)$ must be greater than the minimum threshold (e.g., $\psi_{P}^{t,k}$⩾ 0.5). Otherwise, the system will discard $M_{V_{P}}$ because it is recognized as an untrustworthy message. The result of trust value $\psi_{P}^{t,k}$ will be a new candidate block $\left(\delta_{Block_{i}}\right)$ to be validated in the consensus mechanism. The weighted aggregation of trust value $\psi_{P}^{t,k}$, or the average ratings from the assessors, is defined as the sum of the message credibility $c_{P}^{t,k}$ multiplied by the rating from each assessor $\phi_{P_{A}}$, divided by the number of assessors *A*, as presented in Equation (4).

(4) $$\psi_{P}^{t,k}=\frac{\sum_{i=1}^{A}c_{P}^{t,k}*\phi_{P_{i}}}{A}.$$

#### 3.2.2. Consensus Mechanism & Blockchain Generation

**Figure 2:**
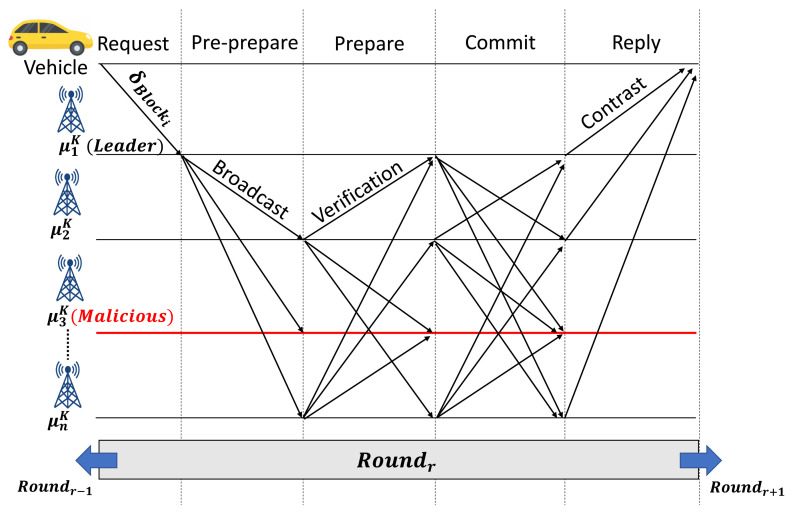
Consensus process in validation block smart contract (VBSC).

A consensus mechanism is utilized to achieve the required agreement between the authorized participant entities to generate a new block transaction into a blockchain network using a particular set of rules. Here, only authorized RSUs are eligible to be the nodes participants (validators) in the consensus mechanism with more extensive storage and computation capability compared to the OBUs. We use the PBFT algorithm to conduct a consensus mechanism due to its advantages, including small resource consumption, high efficiency, consistency, and maturity, making it proper for our proposed scheme. Moreover, PBFT permits the presence of anomalous nodes (f), without changing the consensus decision amongst all of the participating nodes (n), where anomalous nodes are defined as f=(n−1)/3 [33]. Figure 2 illustrates the typical round of PBFT consensus mechanism in blockchain-based IoVEC networks. Several steps of the consensus mechanism are described as follows.

- Leader selection step: In consortium blockchain, RSUs are selected as validators to verify the block $\delta_{Block_{i}}$ transaction in the consensus process. It is assumed that there are *n* edge nodes RSUs $\left(\mu_{n}\right)$ at region *k*, where $\mu^{k}=\left(\mu_{1}^{k},\mu_{2}^{k},\ldots \mu_{n}^{k}\right)$. In each round *r*, a leader is responsible for storing $\delta_{Block_{i}}$ into the blockchain network layer. The leader $\left(\Gamma_{r}\right)$ is chosen among the number of $\mu^{k}$ before the consensus process, and it does not change until after the consensus process.
- Request step: The request step represents a new candidate block generation $\left(\delta_{Block_{i}}\right)$ process in MRSC. After MRSC aggregates $\psi_{P}^{t,k}$, the result of $\delta_{Block_{i}}$ will be validated using consensus mechanism in VBSC.
- Pre-prepare step: In this step, as shown in Figure 2, $\mu_{1}^{k}$ represents the RSU leader $\Gamma_{r}$ that broadcasts $\delta_{Block_{i}}$ to all of the involved nodes $\mu^{k}$ or validators in the consensus mechanism process. Here, the validator nodes $\left(\mu_{2}^{k},\mu_{3}^{k},\ldots \mu_{n}^{k}\right)$ receive $\delta_{Block_{i}}$ using its VBSC.
- Prepare step: Each validator verifies the $\delta_{Block_{i}}$ and broadcasts the message verification among other authorized RSUs $\mu^{k}$. In our scenario, we consider that $\mu_{3}^{k}$ portrays an anomalous node that ignores all validators’ verification request during consensus.
- Commit step: Then, the validators broadcast the *commit* message after receiving over 2f+1 of verification message from other validators in the *prepare-step*.
- Reply step: Finally, the leader $\Gamma_{r}$ proves that the consensus process on round *r* is finished after the consensus reaches over f+1 of the *commit* message and then uploads the verified block $\left(Block_{verified}\right)$ to the blockchain network. Thus, the distributed RSUs automatically obtain the log and authentication of $\delta_{Block_{i}}$, as well as update their ledger, simultaneously. Otherwise, the block $\delta_{Block_{i}}$ will be rejected, and the system starts the next round consensus (r+1).

### 3.3. Blockchain Network Layer

**Figure 3:**
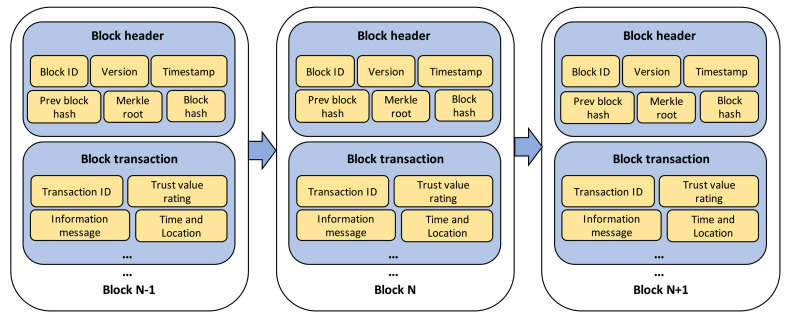
Block structure of transaction.

**Figure 4:**
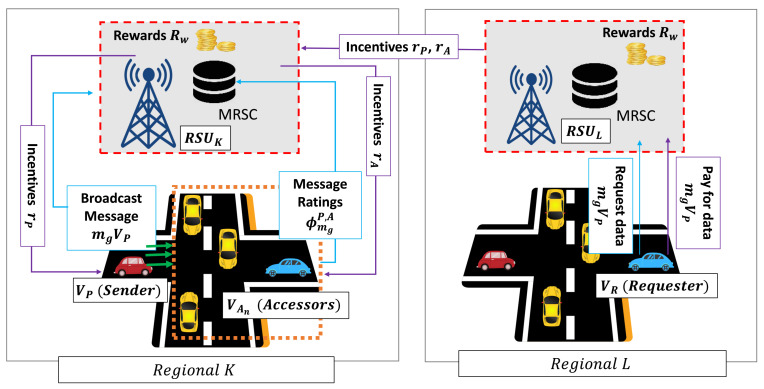
Illustration of incentive mechanism.

Figure 3 shows the structure of transaction blocks, which comprise a block header and block transaction. The block header contains block ID, version, and timestamp as the basic information of block; Merkle root as the hash of the root of Merkle tree structure [34] which is formed by hashing all the recorded transactions; and previous and current block hash used for tracing the information history, as well as proving the validity of transaction block. On the other hand, the block transaction mainly contains the information of the message, which includes transaction ID, trust value rating, and the specific time and location of the event. Additionally, the blockchain network layer is responsible for providing an incentive mechanism based on the ratio of participants’ contribution recorded in MRSC. The incentive is utilized to motivate vehicles to form a trusted data sharing management in IoVEC. $V_{P}$ and $V_{A}$, which correctly provide the road-related messages and assess the message credibility, respectively, will obtain the proportional incentive. Here, the system gives $\left(R_{w}\right)$ reward to the contributing participants $\left(V_{n}\right)$. Let $\chi_{n}$ be the contribution amount of $V_{n}$ and $T_{records}$ be the total of recorded contribution in MRSC. Then, the contributing participants obtain the rewards based on the following calculation:

(5) $$r_{n}=R_{w}\frac{\chi_{n}}{T_{records}},$$

where $r_{n}$ is the reward obtained by $V_{n}$ (i.e., $V_{P}$ and $V_{A}$). In this case, we considered that the $V_{P}$ reward $\left(r_{P}\right)$ is higher rather than that of the $V_{A}$ reward $\left(r_{A}\right)$. Figure 4 illustrates the incentive mechanism scenario, where we assume $V_{P}$ provides valid information about the road-related event in $region_{K}$ with a specific time *t* according to the message credibility assessment from $V_{A}$. On the other region (i.e., $region_{L}$), there is $V_{R}$, which requests the event information of $region_{K}$. $V_{R}$ selects the provided information from $V_{P}$ due to its positive reputation and rating. Then, $V_{R}$ pays a particular incentive to the system to download the data information. Hence, all of the contributing participants (i.e., $V_{P}$ and $V_{A}$) obtain a proportional incentive from the system. As a result, the incentive system enables a trusted data sharing management among vehicles in a decentralized manner.

**Figure 5:**
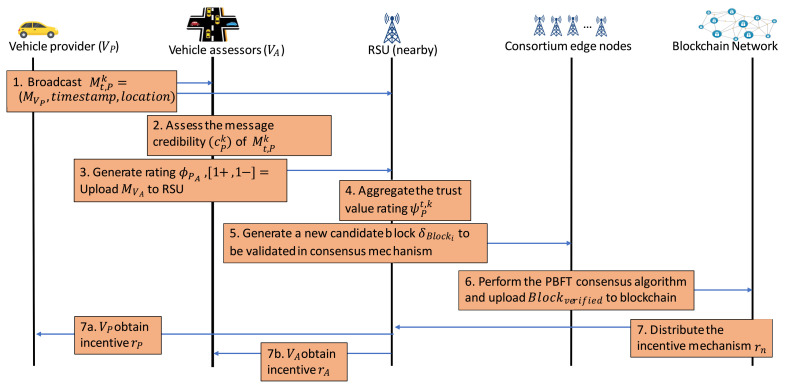
Workflow summary of blockchain-based data sharing management system on Internet of Vehicles edge computing (IoVEC).

Figure 5 summarizes the workflow of the whole framework of trusted data sharing management in IoVEC using blockchain. An information message is broadcasted from the vehicle provider to RSU and the neighboring vehicles as the message assessors. The vehicle assessors evaluate the message credibility by generating ratings and uploaded them to the nearby RSU. Then, the RSU as the edge node aggregates all ratings to obtain the trust value and generates a new candidate block to be validated in the consensus mechanism. The consortium edge nodes perform the PBFT algorithm to validate the candidate transaction block ’s correctness before uploading to the blockchain network. Finally, the blockchain network distributes the incentive based on the vehicle’s contribution in maintaining a trusted data sharing management system in the IoVEC.

## 4. Simulation and Results

### 4.1. System Setup

We designed a trusted data sharing management IoVEC network based on the proposed model, consisting of three layers, i.e., user network layer, blockchain edge layer, and blockchain network layer. Each layer has its setting that distinct from one another. Using the OSMWebWizard package provided in the simulation of urban mobility (SUMO), we modeled a highway traffic scenario to prototype and evaluate IoVEC networks’ efficiency. Here, NS3 as a discrete-event network simulator is used to verify the result, analyzing a trace file for vehicle mobility and message credibility.

**Figure 6:**
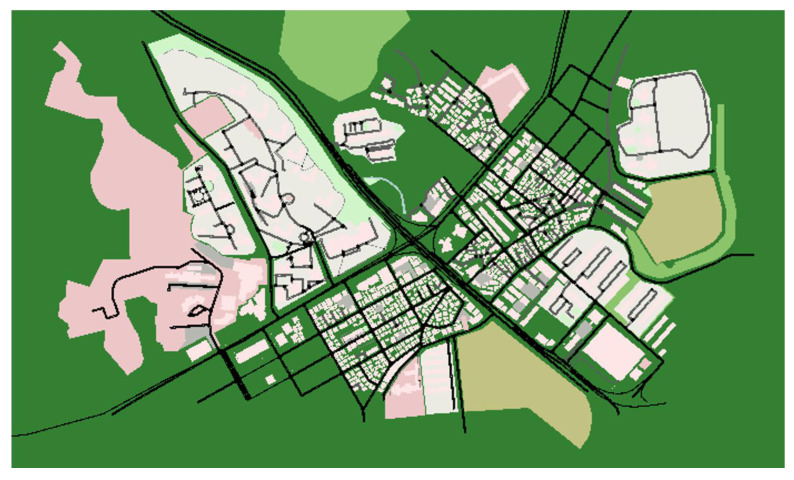
Simulation scenario map.

Figure 6 shows the scenario map that simulates the region of Daeyon in Busan metropolitan city, Republic of Korea. In our study, Hyperledger Sawtooth [35] is utilized to construct a consortium blockchain that supports the PBFT consensus mechanism for its block generation. Furthermore, we leveraged the smart contract feature to form the decentralized incentive scheme to encourage vehicles in the data sharing process. Since Hyperledger Sawtooth allows integration with Ethereum platform [36], our decentralized reward approach is designed using the Ganache CLI-Truffle-Suite interface. The experiments were carried out in Ubuntu 16.04 on Oracle VM VirtualBox, hosted on a personal computer with a CPU Intel(R) Core(TM) i5-4690 CPU @ 3.50 GHz; 3.50 GHz, supported with 16.00 GB RAM. The detailed setting of the simulation is described in Table 2.

### 4.2. Message Credibility

To form an IoVEC, we use an optimized link-state routing protocol (OLSR) as one of the protocol standards in the wireless access for the vehicular environments (WAVE). This protocol enables the system to provide better performance in terms of vehicle mobility, speed, and delay communication [37]. Here, vehicle communication (i.e., V2V and V2I) is supported by DSCR communication standard according to the IEEE 802.11p in the frequency of 5.9 GHz. In this scenario, there are 26 vehicles in the user network layer. However, only ten vehicles are proposed to be neighboring vehicles $V_{A_{n}}$ and placed 50 m apart from the occurred event. Once the vehicle provider $V_{P_{n}}$ broadcasts the road-related message $M_{V_{P}}$ via V2V communication, the neighboring vehicle $V_{A_{n}}$ is allowed to evaluate the message credibility by generating the message rating to edge node. We consider the packet delivery ratio (PDR) as one of the critical parameters for analyzing the performance of IoVEC in terms of message credibility. PDR represents the message credibility ratio that will be aggregated in MRSC of edge node (i.e., RSU).

**Figure 7:**
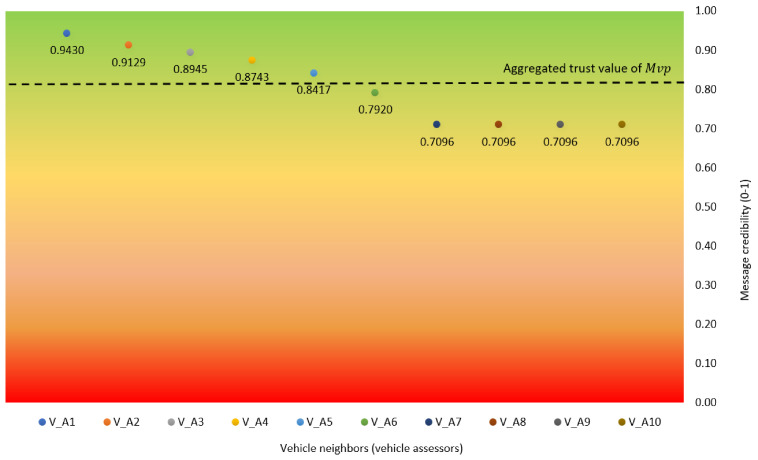
Trust value rating aggregation based on message credibility assessment by ${V_{A}}_{n}$.

Table 3 describes the result of message credibility assessment of $M_{V_{P}}$, whereas Figure 7 shows the trust value rating aggregation based on message credibility assessment over 10 $V_{A}$ on various separations. Green spectrum indicates the highest value of the message’s credibility (i.e., the information is valid). On the contrary, red spectrum indicates the lowest value of the message’s credibility (i.e., the information is false). In short, the result shows that $M_{V_{P}}$ is categorized as trustworthy with trust value rating of 0.8097, as illustrated by the the black dash in Figure 7. This value is obtained from the aggregation of message credibility values from 10 $V_{A}$ on various distances. In this case, each assessor vehicle (i.e., $V_{A_{1}}$ to $V_{A_{10}})$ evaluates the message credibility of $M_{V_{P}}$ and upload the result to the nearby RSU. The vehicle $V_{A_{1}}$ which is closest to the occurred event $M_{V_{P}}$ has the highest trust value with 0.9430. Meanwhile, $V_{A_{7}}$ to $V_{A_{10}}$, whose distance is the farthest to $M_{V_{P}}$, obtain the lowest trust value of 0.7096.

**Figure 8:**
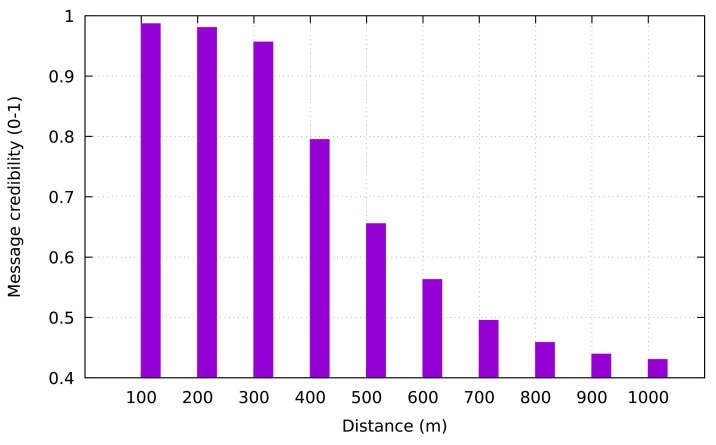
Message credibility rating versus distance of $V_{P}$ to occurred event.

Furthermore, we also observed the relationship between the distance of $V_{P}$ to the occurred event and the message credibility. We placed $V_{P}$ in various distance from the occurred event in the range of 100–1000 m. As shown in Figure 8, the highest value of the message credibility rating comes from the closest distance between $V_{P}$ and $M_{V_{P}}$ (distance of 50 m). Conversely, the lowest value of message credibility is obtained from the highest distance between $V_{P}$ and $M_{V_{P}}$ (distance of 1000 m).

### 4.3. Block Generation

**Figure 9:**
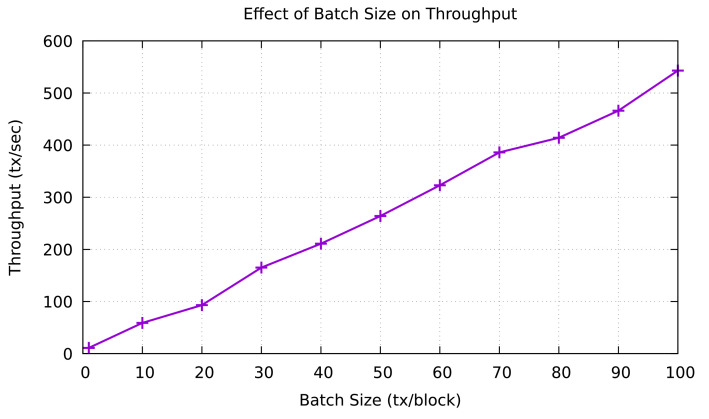
Effect of batch size to throughput [31].

After the MRSC aggregates the trust value rating, its result is then validated using a consensus mechanism in the VBSC consortium blockchain. To construct a consortium blockchain, we use Hyperledger Sawtooth as part of the Hyperledger platform. Hyperledger Sawtooth platform is suitable for our proposed model because it supports the PBFT consensus mechanism equipped by several validators. We utilized Docker containers to facilitate the main core components of Hyperledger Sawtooth architecture, such as transaction processors, validators that represent preselected edge nodes, and Sawtooth Representational State Transfer (REST) server. The consensus mechanism is then conducted by preselected RSUs (validators) to validate a new block transaction. Before that block is stored in the blockchain network layer, the PBFT algorithm requires the agreement over 2f+1 among the participating nodes in the consensus process. Moreover, Hyperledger Sawtooth offers the batch size that represents the block with many transactions involved. Figure 9 presents the effect of batch size on throughput. We evaluate this scenario by running multiple tests to change the batch size up to 100 tx/block. As a result, we can see that the throughput on various batch sizes increases linearly and reaches 543 tx/sec at the total batch size of 100 tx/block.

### 4.4. Distributed Incentive Model

To support an adequate incentive for the information provider (vehicle ${V_{P}}_{n}$), we implemented Ethereum smart contracts as a decentralized and tamper-proof incentive mechanism. Each vehicle in various regions broadcasts the respective road-related message to the RSU with a varying amount of information. After the message $M_{V_{P}}$ is validated using blockchain consortium, ${V_{P}}_{n}$ promptly receives a certain amount of incentive whenever another party (RSU and other vehicles) uses ${V_{P}}_{n}$’s information. The incentive distributed is in Ether, with a linear amount with the information provided by ${V_{P}}_{n}$. The exact amount of Ether to the data size can be freely adjusted by the system as required.

We utilized a smart contract feature in the Ethereum platform through Ganache Truffle (v.2.4.0) graphical user interface (GUI). The default setting is applied where the gas limit is set to be 6,721,975 units, with the gas price is 20,000,000,000 wei running on a remote procedure call (RPC) server HTTP://127.0.0.1:7545 (accessed on 11 January 2021) with an auto mining mode. The address for all vehicles is derived from Ganache that holds 100.00 ETH each (publicly available). In a real-world implementation, the address does not depend on a single user interface, and the address is managed in the private wallet of each entity.

We performed the incentive distribution with a different number of vehicles (the message providers) ${V_{P}}_{Kn}-RSU_{n}$ with a specific number of $M_{V_{P}}$ sizes together with their respective RSUs for ease of presentation. Experiments were carried out (Exp. 1 and Exp. 2) with the main objective of knowing the total number of gas usage units (units) in distributing incentives for the message providers. The incentives for each ${V_{P}}_{Kn}$ are differentiated by the amount of valid data provided by the respective vehicles. For the first five experimental sequences, vehicles ${V_{P}}_{K1}-{V_{P}}_{K5}$ are connected to $RSU_{1}$, which is in regional K. Meanwhile, for the last five experimental sequences, vehicles ${V_{P}}_{L1}-{V_{P}}_{L5}$ are connected to $RSU_{2}$ in regional L as shown in Table 4.

**Figure 10:**
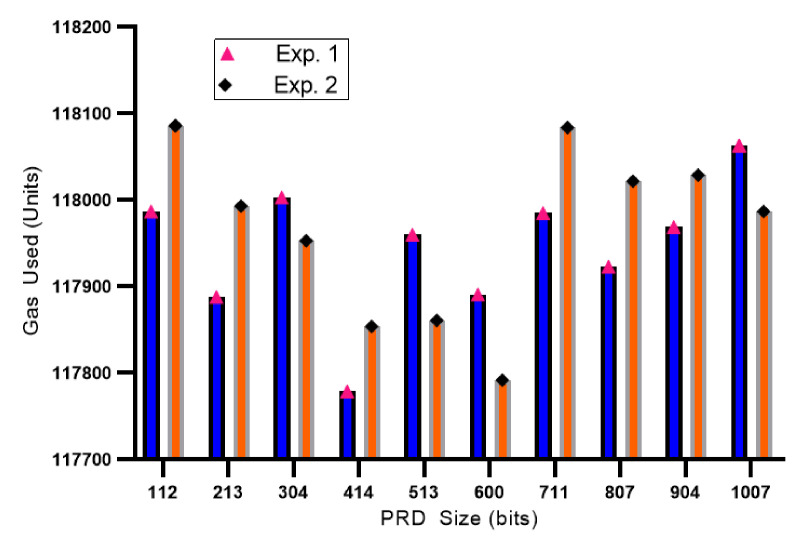
The information on gas usage by RSU in distributing Ether for ${V_{P}}_{Kn}$.

Table 4 presents the performance of the total gas usage and Ether distribution for the message provider. The message sizes are sorted from least to largest size. In the Exp. 1 GU (gas usage), the average gas usage by the RSU to distribute incentives was 117,944 units, with the minimum usage recorded at 117,779 units (414 bits) in ${V_{P}}_{K4}-RSU_{1}$, and the largest usage was 118,063 units (304 bits) in ${V_{P}}_{K3}-RSU_{1}$. Meanwhile, on Exp. 2, the recorded average use of gas was 117,966 units, with the minimum usage was 117,792 units (${V_{P}}_{L1}-RSU_{2}$ with 600 bits of PDR size), and the largest usage of gas was recorded at 118,086 units (${V_{P}}_{K1}-RSU_{1}$). Furthermore, Figure 10 illustrates the information on gas usage by RSU in distributing Ether for the contributed vehicles. Even though Figure 10 shows the amount of gas usage is significant from one another, the amount of gas difference between transactions is relatively the same by using units notion. Smart contracts store the address information of the requester and provider, while PDR data is stored off-chain. The Ethereum network only stores arbitrary values of related information.

## 5. Discussion

Recently, blockchain has been widely studied to form a secure, trusted, and decentralized data management in the vehicular network. Several works have proposed blockchain-based IoV solutions to protect road-related information sharing among vehicles to improve traffic safety and efficiency [2,15,16]. Here, our proposed system combines these solutions’ advantages to develop a decentralized trusted data sharing management in IoVEC. Table 5 shows the key parameter comparison of our proposed system with other solutions by emphasizing the five distinguished parameters: type of blockchain, implementation of MEC technology, design of message credibility assessment, type of consensus algorithm, and implementation of incentive mechanism in the system.

Compared to the proposals by Yang et al. [2] and Zhang et al. [16] which adopt conventional vehicular network architectures, our proposed model employs a combination of MEC technology and IoV to achieve better performance in data storage and sharing. Our proposed model, i.e., IoVEC, also aims to improve user experience by allowing the computing process be closer to the vehicles. Hence, it can reduce service delay and transmission latency while also enhancing QoS.

Kang et al. [15] also make use of MEC technology. However, they do not make use of message credibility assessment, which can protect the system from malicious vehicles that try to sabotage the system by transmitting wrong information (i.e., spoofing attack). On the other hand, our system allows the neighboring vehicle to evaluate the received messages and generate a credibility rating, which helps in evading spoofing attacks. Furthermore, no incentive scheme is present in their model. In this case, vehicles may not be interested in sharing the information to the system since no apparent benefit are received. In contrast, our incentive mechanism can encourage vehicles to contribute, thus promoting information sharing within the system.

Furthermore, we employ consortium blockchain to avoid SPoF attacks in a centralized system and prevent data modification attacks that may broadcast and create a modified block transaction in the consensus process conducted by compromised RSUs. Compared to other works which mainly use a joint PoW and PoS mechanisms (i.e., References [2,15,16]) we utilize PBFT consensus mechanism which is better in ensuring the system’s consistency and guarantee that the new validated block will be distributed to all nodes in the system. Hence, all the legitimate nodes have a consistently updated database in the IoVEC network. Additionally, to support an adequate incentive mechanism for the information provider and assessors, we implement Ethereum smart contract as a decentralized and tamper-proof incentive mechanism. This incentive is aimed to encourage vehicle participation in maintaining the system’s reliability and sustainability.

By leveraging the several advantages of our proposed framework as discussed above, the blockchain-based IoVEC framework can be utilized to address the limitation of ITS application (e.g., connected cars application), especially in terms of enhancing system performance and security protection. In IoVEC, MEC can improve the system performance; providing low latency, high bandwidth, and real-time communication by placing its server in the edge network to be closer to the user. Furthermore, the blockchain can form a decentralized and trusted data management system, as well as to provide the users with privacy and security. Nevertheless, future discussions are encouraged to address relevant issues in the blockchain-based IoVEC. For instance, the construction of a robust message authentication mechanism in order to strengthen privacy and security protection in the blockchain-based IoVEC would be of great importance. In Reference [17], the authors explain several techniques that might be adequate to be implemented for message authentication mechanisms, such as ring signature, attribute-based encryption, secure multi-party encryption, homomorphic encryption, group signature, and trusted execution environment (TEE)-based solution. Furthermore, the blockchain scalability issue still needs to be considered in the real-world blockchain-based application implementation. Several works have proposed solutions to overcome these issues, such as leveraging layer-two protocols with off-chain transaction [38], sharding [39], and alternative blockchain consensus architectures [40]. We consider these challenges as part of our future research.

## 6. Conclusions

We have introduced a consortium blockchain and smart contracts to achieve a decentralized trusted data sharing management system in IoVEC. In this paper, smart contracts are exploited to accomplish an efficient, reliable, and secure data management system. Here, two smart contracts, MRSC and VBSC, are employed and placed on RSUs as the distributed edge network infrastructure. MRSC is used to collect and aggregate the trust value rating, while VBSC performs the consensus mechanism. Furthermore, this framework permits vehicles to validate the credibility of messages from their neighboring vehicles by generating a reputation rating. Additionally, we utilized an incentive mechanism based on Ethereum smart contract to motivate and propel the vehicles to contribute and sincerely share their data to obtain certain rewards from the system. Packet delivery ratio, considered to represent trust value rating of data sharing efficiency in IoVEC-Blockchain, shows a favorably positive performance and feasible to form a decentralized trusted data management system. Lastly, further studies are still required to apply a robust message authentication mechanism and cope with the blockchain scalability issue.

## Figures and Tables

**Table 1:** Summary of notations.

Symbol	Description
$V_{i}$	The legitimate vehicle in the network
$PK_{V_{i}}$,$SK_{V_{i}}$	The vehicle’s public and private key pair
TP	The trusted party for managing vehicle enrollment
$Cert_{V_{i}}$	The vehicle’s corresponding certificate
$V_{P}$	The provider vehicle that share the road-related message
$V_{A}$	The assessor vehicle that assess the road-related message from $V_{P}$
$M_{i}$	The road-related message from the legitimate vehicle $V_{i}$
$M_{V_{P}}$	The information message from $V_{P}$
$M_{t,P}^{k}$	The collected information message by $V_{P}$ in *t* time and location *k*
$c_{p}^{k}$	The credibility of the message $M_{t,P}^{k}$
$M_{V_{A}}$	The message credibility assessment uploaded by $V_{A}$
$\phi_{P_{A}}$	The message rating of $V_{P}$ generated by $V_{A}$
$\delta_{Block_{i}}$	The new block candidate that will be validated in consensus mechanism
$\psi_{P}^{t,k}$	The aggregated trust value of $M_{V_{P}}$ according to $\phi_{P_{A}}$

**Table 2:** Simulation parameters of IoVEC.

Parameter	Value
number of Vehicles	26
vehicle speed	20 m/s
WAVE ITS band	5.9 GHz
MAC type	IEEE 802.11p
physical mode	OFDM (6 Mbps rate)
the type of routing protocol	OLSR
transmission rate	2.048 Kbps
the model of propoagation loss	Two-ray ground
fading model	Nakagami fading
data rate	6 Mbps
power transmission	20 dBm
packet interval	100 ms
data rate	6 Mbps
channel bandwidth	10 MHz
antenna height	1.5 m
simulation time	100 s

**Table 3:** Result of message credibility assessment of $M_{V_{P}}$.

Vehicle Neighbors	Distance from $M_{V_{P}}$	Message Credibility [0–1]	Accuracy Reduction [0–1]
$V_{A_{1}}$	50 m	0.9430	0.0570
$V_{A_{2}}$	100 m	0.912905	0.0871
$V_{A_{3}}$	150 m	0.89447	0.1055
$V_{A_{4}}$	200 m	0.874277	0.1257
$V_{A_{5}}$	250 m	0.841686	0.1583
$V_{A_{6}}$	300 m	0.791951	0.2080
$V_{A_{7}}$	350 m	0.709643	0.2904
$V_{A_{8}}$	400 m	0.709643	0.2904
$V_{A_{9}}$	450 m	0.709643	0.2904
$V_{A_{10}}$	500 m	0.709643	0.2904

**Table 4:** Performance of the total gas usage and Ether distribution for the packet delivery ratio (PDR) provider.

Vehicle (Message Provider)	Message Size	Incentive (Ether)	Exp. 1 GU (Units)	Exp. 2 GU (Units)	Regional
${V_{P}}_{K1}-RSU_{1}$	112 bits	0.0001	117,987	118,086	L→ K
${V_{P}}_{K2}-RSU_{1}$	213 bits	0.0002	117,888	117,993	L→ K
${V_{P}}_{K3}-RSU_{1}$	304 bits	0.0003	118,003	117,953	L→ K
${V_{P}}_{K4}-RSU_{1}$	414 bits	0.0004	117,779	117,854	L→ K
${V_{P}}_{K5}-RSU_{1}$	513 bits	0.0005	117,960	117,861	L→ K
${V_{P}}_{L1}-RSU_{2}$	600 bits	0.0006	117,891	117,792	K→ L
${V_{P}}_{L2}-RSU_{2}$	711 bits	0.0007	117,985	118,084	K→ L
${V_{P}}_{L3}-RSU_{2}$	807 bits	0.0008	117,923	118,022	K→ L
${V_{P}}_{L4}-RSU_{2}$	904 bits	0.0009	117,969	118,029	K→ L
${V_{P}}_{L5}-RSU_{2}$	1007 bits	0.001	118,063	117,987	K→ L

**Table 5:** The key parameter comparison of our proposed system with other solutions.

Key Parameter/Technology	Yang et al. [2]	Kang et al. [15]	Zhang et al. [16]	Proposed System
Blockchain type	Public	Consortium	Consortium	Consortium
MEC technology	No	Yes	No	Yes
Message credibility assessment	Yes	No	No	Yes
Consensus mechanism	Joint PoW and PoS	Joint PoW and PoS	Joint PoW and PBFT	PBFT
Incentive scheme	No	No	No	Yes

## Data Availability

Not applicable.

## Abbreviations

The following abbreviations are used in this manuscript:

IoV	Internet of Vehicle
ITS	Intelligent Transportation System
VANET	Vehicular Ad hoc Network
IoVEC	IoV Edge Computing Network
SPoF	Single Point of Failure
MEC	Mobile Edge Computing
PBFT	Practical Byzantine Fault Tolerance
RSU	Roadside Unit
OBU	Onboard Unit
V2I	Vehicle to Infrastructure Communication
V2V	Vehicle to Vehicle Communication
TP	Trusted Party
DSRC	Dedicated Short-Range Communication
MRSC	MEssage Record Smart Contract
VBSC	Validation Block Smart Contract
WAVE	Wireless Access for Vehicular Network
SUMO	Simulation of Urban Mobility
MAC/PHY	Medium Access Control/Physical Layer
OLSR	Optimized Link-State Routing
PDR	Packet Delivery Ratio
